# Obesity-Associated Non-T2 Mechanisms in Obese Asthmatic Individuals

**DOI:** 10.3390/biomedicines11102797

**Published:** 2023-10-16

**Authors:** Harshita Shailesh, Ajaz A. Bhat, Ibrahim A. Janahi

**Affiliations:** 1 Department of Medical Education, Sidra Medicine, Doha 26999, Qatar; hshailesh@sidra.org; 2 Precision Medicine in Diabetes, Obesity and Cancer Research Program, Department of Human Genetics, Sidra Medicine, Doha 26999, Qatar; abhat@sidra.org; 3 Department of Pediatric Medicine, Sidra Medicine, Doha 26999, Qatar; 4 Department of Pediatrics, Weill Cornell Medicine, Doha 24144, Qatar

**Keywords:** obesity, asthma, hyperinsulinemia, microbiome

## Abstract

Obesity and asthma are two common health issues that have shown increased prevalence in recent years and have become a significant socioeconomic burden worldwide. Obesity increases asthma incidence and severity. Obese asthmatic individuals often experience increased exacerbation rates, enhanced airway remodeling, and reduced response to standard corticosteroid therapy. Recent studies indicate that obesity-associated non-T2 factors such as mechanical stress, hyperinsulinemia, systemic inflammation, adipose tissue mediators, metabolic dysregulation, microbiome dysbiosis, and high-fat-diet are responsible for increased asthma symptoms and reduced therapeutic response in obese asthmatic individuals. This manuscript reviews the recent findings highlighting the role of obesity-associated factors that contribute to airway hyper-reactivity, airway inflammation and remodeling, and immune cell dysfunction, consequently contributing to worsening asthma symptoms. Furthermore, the review also discusses the possible future therapies that might play a role in reducing asthma symptoms by diminishing the impact of obesity-associated non-T2 factors.

## 1. Introduction

Asthma is one of the most prevalent non-communicable lung diseases that impact both pediatric and adult populations globally. An international study reported that approximately 300 million people worldwide are affected by asthma, with around 1000 asthma-related deaths every day [1]. Asthma is characterized by airway limitation due to a combination of pathophysiological events, including airway obstruction, hyper-reactivity, inflammation, increased mucus production, and airway remodeling [2].

The prevalence of obesity is increasing at an alarming rate worldwide. Extensive studies over the last two decades have shown that increased adiposity is linked to the risk of asthma incidence in children [3,4,5,6]. A recent meta-analysis revealed that obesity can increase the likelihood of asthma by 50% in children [7]. Another study based on the data from the Taiwan Children’s Health Study informed that an increase in adiposity before the age of 6 years is linked to an enhanced risk of childhood asthma, while adiposity gain in the prepubertal stage predicts asthma in young adulthood [8]. Individuals who are obese and suffer from asthma often face a more severe manifestation of exacerbations and a diminished quality of life than their normal-weight counterparts diagnosed with asthma [9]. This disparity is further highlighted by obese asthmatic patients’ reduced responsiveness to conventional asthma treatments. Such limited effectiveness not only undermines these individuals’ wellbeing and health prospects but also imposes an additional societal burden. This situation underscores the imperative for tailored, effective intervention strategies for obese individuals with asthma, ensuring improved and equitable health outcomes for all asthma patients [9,10]. 

**Figure 1:**
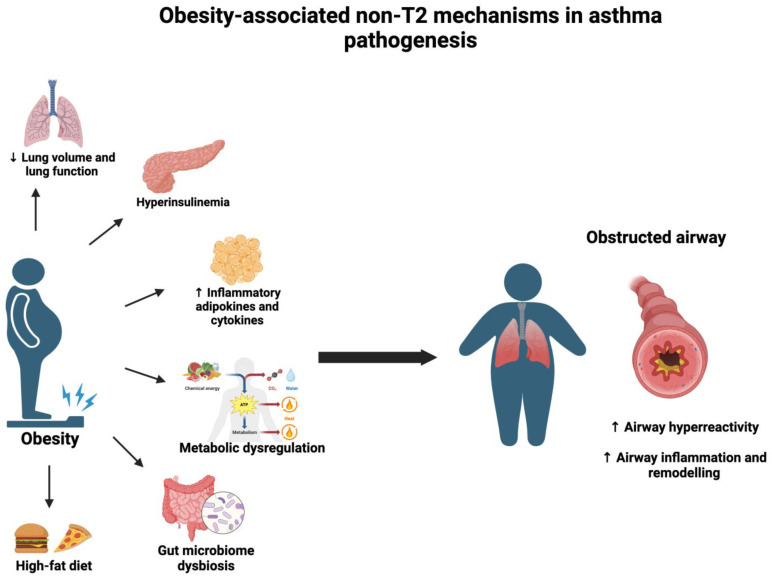
Obesity-associated factors that contribute to asthma development and severity. Obesity-associated characteristic features such as reduced lung volumes and function, increased proinflammatory cytokines and adipokines, hyperinsulinemia, dysregulation in metabolism, dysbiosis in gut microbiome, and consumption of high-fat diet promote asthma phenotype in individuals. These changes play an important role in increasing airway hyper-responsiveness and modulating immune response, resulting in inflammation and remodeling of the airway and poor response to standard asthma therapy. ↑—increase; ↓—decrease.

The intricate and bidirectional relationship between obesity and asthma has been a focal point of contemporary research, delineating the mutual impact they exert on each other. The predisposition of obesity in contributing to the onset of asthma and influencing its pathobiology is realized through diverse mechanisms. Within the obese population, an escalation in abdominal adiposity leads to the upward displacement of the diaphragm, culminating in a reduction in total lung volume and a diminished expiratory reserve volume, subsequently inducing airway resistance [11]. Additional obesity-induced factors play a substantial role in exacerbating asthma symptoms. Insulin resistance, metabolic alterations, immune dysregulation, and microbiome dysbiosis stand as significant contributors to airway hyper-responsiveness, pervasive lung inflammation, and comprehensive airway remodeling, the hallmarks of asthma [12,13,14,15] (Figure 1) (Table 1).

Conversely, the management strategies for asthma, which frequently employ chronic inhaled and intermittent oral glucocorticoids, inadvertently foster the development of obesity [16]. The resultant uncontrolled asthma further imposes limitations on physical activity and aerobic exercise endurance, thereby exacerbating obesity [17] This comprehensive review encapsulates findings from numerous recent studies that probed the role of non-T2 factors associated with obesity in promoting asthma pathogenesis. It explores diverse research mediums, including in vitro studies, animal models, ex vivo investigations utilizing human lung tissues, and retrospective data from obese asthmatic individuals across varied cohorts. Beyond delineating the interplay between obesity and asthma, this review anticipates the future by spotlighting the promising therapeutic potential of anti-diabetic drugs in mitigating obesity-associated asthma, offering a novel approach towards more holistic and effective treatment paradigms.

**Table 1:** Major studies on the impact of obesity-associated non-T2 factors including altered lung mechanics, hyperinsulinemia, metabolic alterations, immune dysregulation, microbiome dysbiosis, and high-fat diet on asthma pathogenesis.

Obesity-Associated Non-T2 Factors	Type of Study	Source	Key Findings	Authors
Adiposity-associated changes in lung mechanics	In humans	Retrospective study of 373 adults with different BMI	↑ BMI is associated with exponential reduction in FRC and ERV	Jones et al., 2006 [18]
	In humans	Cross-sectional study of healthy adults	↑ WC and WHR are associated with noticeable changes with volume, capacity, and mechanics of the lung	Shanmugasundaram et al., 2023 [19]
	In humans	Retrospective study from mild to moderate obese asthmatic children of Childhood Asthma Management Program (CAMP)	↑ BMI is associated with increase in FEV1 and FVC and decrease in FEV1/FVC ratio	Tantisira et al., 2003 [20]
Hyperinsulinemia	Ex vivo	SMCs from bovine trachea	Hypercontractilibility of SMCs	Gosens et al., 2003 [21]
	Ex vivo	SMCs from bovine trachea	↑ Accumulation of smooth muscle myosin and laminin	Dekkers et al., 2009 [22]
	In vitro Ex vivo	Human ASMs Trachea of high-fat-diet-fed mice	↓ β-AR agonist-mediated relaxation of airway smooth muscle cells	Xu et al., 2020 [23]
	In vivo Ex vivo	Diet-induced obese mice Human tracheal smooth muscle strips	↑ Vagus nerve-stimulated airway hyper-responsiveness by inhibiting M2 muscarinic receptors ↑ Contraction of trachial smooth muscle cells in response to electric field stimulation by inhibiting M2 muscarinic receptors	Nei et al., 2014 [24]
	In vivo	Diet-induced obese mice	Reducing insulin levels decreases vagus nerve-stimulated airway hyper-responsiveness by restoring M2 muscarinic receptor function	Proskocil et al., 2021 [25]
	In vivo	Diet-induced obese mice	↑ cholinergic nerve activation and airway hyper-responsiveness	Leiria et al., 2014 [12]
	In vivo	Diet-induced obese mice	↑ sensory nerve population in the lung	Calco et al., 2022 [26]
	In vivo	Diet-induced obese mice	↑ TGF-β1 expression in the lung, fibrosis of the lung, and airway hyper-responsiveness	Park et al., 2019 [27]
	In humans	Retrospective data from obese children	↑ increased risk of allergic asthma in obese children	Sánchez Jiménez et al., 2014 [28]
	In humans	Retrospective data from National Health and Nutrition Examination Survey (NHANES), United States	Insulin resistance increases the risk of asthma incidence in obese individuals in a dose-dependent manner, independent of hypertension, systemic inflammation, hyperglycemia, and hypertriglyceridemia	Cardet et al., 2016 [29]
	In human	Retrospective cohort of US adults with obesity and asthma	Prediabetes and diabetes states increase asthma exacerbation rate by 27% and 33%, respectively	Wu et al., 2019 [30]
Inflammation	In humans	Obese asthmatic women vs. obese women (in adults)	↑ leptin increases systemic inflammation and airway reactivity without lung inflammation in obese asthma	Sideleva et al., 2012 [31]
	In vitro	Normal human lung fibroblasts	leptin promotes the expression of MCP-1, eotaxin, IP-10, IL-6, and IL-8	Watanabe et al., 2019 [32]
	In vitro	Human primary bronchial epithelial cells	Leptin promotes the expression of ICAM-1, CCL11, VEGF, IL-6, and G-CSF	Suzukawa et al., 2015 [33]
Metabolic dysregulation	In vivo	Diet-induced obese mice	Changes in metabolomic profile of lung with altered concentration of 1C and TCA cycle metabolites	Rautureau et al., 2021 [34]
	In humans	Obese asthma vs. lean asthma (in adults)	Changes in metabolomic profile of serum, sputum, and PBMCs. Altered metabolomics are associated with airway inflammation	Liu et al., 2018 [35]
Microbiome dysregulation	In humans	Obese asthma, obese non-asthma, non-obese asthma, and healthy control (in adults)	Obesity and asthma synergistically contribute to inflammation and microbiome alteration	Michalovich et al., 2019 [36]
High-fat diet	In vivo	Diet-induced obese mice vs. normal-diet-fed mice	high-fat diet increases AHR, serum IgE, and lung TNF-α upon OVA challenge	Kim et al., 2015 [37]
	In humans In vivo	Obese asthmatics vs. lean asthmatics (in adults) High-fat diet induced obese mice vs. normal-diet-fed mice	Increased levels of palmitic acid and DPP4 in the BALF of obese asthmatics as compared to lean asthmatics High-fat-diet-associated increase in palmitic acid increases eosinophils, neutrophils, and eotaxin -2 in BALF upon allergic trigger	Dimasuay et al., 2023 [38]

↑—increase; ↓—decrease. Abbreviation: BMI: body mass index; FRC: functional residual capacity; ERV: expiratory reserve volume; WHR: waist-to-hip ratio; WC: waist circumference: SMC: smooth muscle cell; ASM: airway smooth muscle cell; FEV1: forced expiratory volume in 1 s; FVC: forced vital capacity; β-AR: β-Adrenergic receptor; TGF-β1: transforming growth factor-β1; MCP-1: monocyte chemoattractant protein-1; IP-10: interferon-inducible protein 10; IL: interleukin; ICAM-1: intercellular adhesion molecule; CCL11: C-C motif chemokine 11; VEGF: vascular endothelial growth factor; G-CSF: granulocyte-colony stimulating factor; 1C: 1 carbon; TCA: tricarboxylic acid; PBMC: peripheral blood mononuclear cells; AHR: airway hyper-reactivity; IgE: immunoglobulin E; TNF-α: tumor necrotic factor-α; OVA: ovalbumin; DPP4: dipeptidyl peptidase 4; and BALF: bronchoalveolar lavage fluid.

## 2. Methodology

We conducted a comprehensive literature search focused on extracting relevant articles that discuss the diverse aspects and connections between obesity and asthma as well as their impact on lung mechanics and function. Our search was confined to the PubMed database to ensure the inclusion of credible and peer-reviewed articles in our review. The literature search encompassed articles published over a span of more than four decades, from the year 1980 up to the present year, 2023. A meticulous search was conducted using the following key terms, either singly or in combination: “Asthma, Obesity, Lung mechanics, Lung function, Hyperinsulinemia and lung, Obesity and inflammation, Obesity, microbiome, and asthma, Obesity and metabolic dysregulation, Diet, obesity, and asthma, Antidiabetic therapy and asthma”. Each search term was carefully selected to encompass the broad spectrum of obesity-associated factors and their interplay with asthma. Beyond the database search, additional articles were identified by manually examining the reference lists of pertinent review articles and primary research papers. This method ensured the inclusion of potential articles that might have been missed in the initial database search, adding depth and breadth to our literature review. We included both human and animal studies to provide a comprehensive overview of the topic. Articles were excluded if they did not focus on the intersection between obesity and asthma or if they did not contribute substantial information regarding lung mechanics, function, or associated factors mentioned in the search terms.

## 3. Obesity and Lung Mechanics

Several studies have described the direct effects of obesity on the respiratory system. For example, Jones et al., 2006, retrospectively investigated the impact of BMI on lung function using data from pulmonary function tests of 373 patients [18]. They found that increase in BMI was associated with exponential decrease in functional residual capacity and expiratory reserve volumes [18]. Furthermore, an increased waist-to-hip ratio shows a positive correlation with higher airway resistance, area of reactance, resonant frequency, and reduced reactance in females [19]. Similarly, increased waist circumference in these females is associated with enhanced airway resistance, airway reactance, resonant frequency, and decreased ratio of forced expiratory volume in 1 s (FEV1)/forced vital capacity (FVC) [19]. On the other hand, obesity has different impact on lung function in pediatric population, where increase in weight is associated with elevated FEV1 and FVC but with reduced FEV1/FVC ratio due to airway dysanapsis [20,39].

Several mechanisms that link obesity and reduced airway mechanics are explored. Obesity-associated accumulation of adiposity in the mediastinum and abdominal cavities reduces the compliance of the lung, respiratory system, and chest wall, eventually contributes to the development of respiratory symptoms such as altered breathing pattern, wheezing, and dyspnea [40,41]. In addition, increased pleural pressure in obesity also contributes to the narrowing and closure of the airway and enhanced resistance in the respiratory system [42]. Reduced lung volumes and increased airway resistance are linked to airway hyper-responsiveness in obese individuals, which is further exaggerated in obese asthmatic individuals [43]. Weight loss by surgery, diet, or exercise can reverse airway hyper-responsiveness, improve function of the lung in asthmatic individuals with obesity and reduce their asthma symptoms [15,44,45].

## 4. Obesity-Associated Hyperinsulinemia and Airway Hyper-Responsiveness

**Figure 2:**
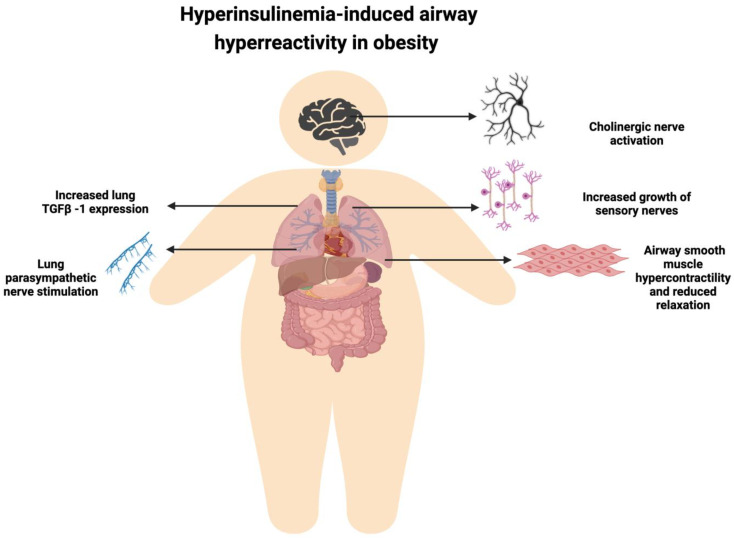
Hyperinsulinemia-induced airway hyper-reactivity in obesity: obesity-associated hyperinsulinemia can promote airway hyper-reactivity via several mechanisms such as activation of brain cholinergic nerves, increased growth of lung sensory nerves, enhanced contractability of airway smooth muscle cells, increased expression of TGF-β1, and stimulation of lung parasympathetic nerves.

Several extensive epidemiological and cross-sectional studies have shown a dose-dependent relationship between obesity and hyperinsulinemia [46]. Hyperinsulinemia is considered one of the most critical non-T2 factors that can promote asthma pathogenesis through multiple mechanisms in the context of obesity (Figure 2) [24,47,48]. Gosens et al., 2003. showed for the first time that insulin exposure induces hypercontractile phenotype of smooth muscle cells isolated from bovine trachea [21]. In a follow-up study, the same group further investigated the mechanism through which insulin induces contractile phenotype in airway smooth muscle cells (ASMs). Here, authors showed that exposure to insulin enhances the accumulation of contractile proteins including sm-myosin and calpolin in bovine tracheal smooth muscle cells in vitro and sm-myosin protein in bovine tracheal smooth muscle strips in organ bath. RT-PCR results further confirmed that insulin treatment increases the mRNA levels of sm-MHC, a contractile phenotype marker of ASMs. Using specific inhibitors, the authors demonstrated that insulin increases the levels of sm-myosin and calpolin by activating Rho-kinase and PI3-kinase signaling in ASMs [22]. In addition to inducing contraction, hyperinsulinemia also modulates β-adrenergic receptor (β-AR)-agonist-mediated relaxation of airway smooth muscle cells [23]. β-AR agonists serve as bronchodilators due to their ability to induce relaxation of airway smooth muscle cells. These agonists bind to β2-AR and induce bronchodilation via cAMP production. Furthermore, the phosphodiesterase family protein, PDE4, acts as a key regulator of this pathway by causing the degradation of cAMPs. However, pre-treatment with insulin reduces the ability of airway smooth muscle cells to produce cAMP in response to the β2-AR agonist, isoproterenol, leading to reduce relaxation. Mechanistically, insulin treatment induces phosphorylation of β2-AR at the G protein-coupled receptor kinase protein site (serine 355/356) leading to the uncoupling of Gs protein and binding to Gi protein. Furthermore, this insulin-mediated GRK2 phosphorylation promotes the ERK1/2 phosphorylation cascade, subsequently inducing phosphorylation of PDE4D, resulting in its activation. These findings were further confirmed by authors using high-fat-fed obese mice [23]. Collectively, these results indicate the substantial role of obesity-associated hyperinsulinemia in inducing bronchoconstriction and poor response to conventional asthma therapy in obese asthmatic patients.

Sensory nerves in the airway epithelium respond to external stimuli in the lumen and transduce the signal to the central nervous system, consequently triggering the parasympathetic nerves in the airway [49]. Activated parasympathetic nerves release the acetylcholine (Ach) neurotransmitter that binds to the M_3_ muscarinic receptor located on the airway smooth muscle cells and induces their contraction. In a feedback mechanism, M_2_ muscarinic receptors located on the parasympathetic nerves inhibit the release of Ach and reverse bronchoconstriction [26,50]. Dysregulation in this lung–brain axis is often associated with airway hyper-reactivity, a characteristic feature of asthma. 

Several studies indicate that increased insulin levels can induce airway hyper-responsiveness by stimulating vagus (parasympathetic) nerves. Using a polygenic model of diet-induced obesity in rats, Nei et al., 2014, systematically showed that insulin can enhance vagus-nerve-stimulated airway hyper-responsiveness independent of lung inflammation and remodeling [24]. In their study, the authors showed that obese-prone rats fed with a high-fat diet had increased insulin levels and enhanced bronchoconstriction in response to vagus nerve stimulation as compared to obese-prone rats on a low-fat diet or obese-resistant rats on low-/high-fat diet. However, intraperitoneal application of streptozotocin (STZ), an inhibitor of insulin secretion, to the high-fat-fed obese-prone rats reduced their ability to potentiate bronchoconstriction after vagus nerve stimulation, which was reversed by restoring insulin levels to pre-STZ treatment level by acute or chronic insulin treatment. Of note, the inhibitor did not affect bronchoconstriction in obese-resistant rats on a high-fat diet [24].

On the other hand, insulin treatment in these rats was able to potentiate parasympathetically induced bronchoconstriction. In the same study, authors also demonstrated that insulin could potentiate airway hyper-reactivity by inhibiting M_2_ muscarinic receptors in high-fat-diet fed obese prone rats. Similarly, ex vivo studies also showed that insulin treatment increases the contraction of isolated human trachea upon electric field stimulation by inhibiting M_2_ muscarinic receptors [24]. A sequel to this, a study from the same group showed that reducing insulin in diet-induced obese mice decreases airway hyper-reactivity by restoring M_2_ receptor function. Here, authors showed that treating with pioglitazone, a nuclear receptor peroxisome proliferator-activator receptor-γ (PPAR-γ) agonist, reduces hyperinsulinemia in high-fat-diet-fed mice without affecting body weight and fat gain. Subsequently, these mice showed reduced airway hyper-reactivity by improving M_2_ receptor function [25]. Conclusively, these studies indicated that obesity-mediated hyperinsulinemia has a significant role in augmenting airway hyper-responsiveness by dysregulating the function of lung parasympathetic nerves.

A mechanistic study in vivo by Leiria et al., 2015, indicated that hyperinsulinemia in obesity can promote airway hyper-reactivity by directly activating cholinergic neurons in the brain stem [12]. Here, the authors showed that as compared to the wild-type mice, the high-fat-fed obese mice exhibit elevated airway hyper-reactivity (AHR) that is independent of lung inflammation. Furthermore, enhancing central insulin levels via intracerebroventricular (ICV) injection increases AHR without increasing peripheral insulin levels in wild-type mice. These changes were not observed in vesicle acetylcholine transporter knockdown (VAChT KDHOM^−/−^) mice and bilateral cervical vagotomized (VX) control mice. Furthermore, using specific inhibitors or siRNA-mediated knockdown experiments, authors revealed that hyperinsulinemia stimulates cholinergic neurons by activating the ERK-signaling pathway. Immunohistochemistry results confirmed that insulin activates the ERK-signaling cascade in the cholinergic neurons located in the dorsal motor nucleus of the vagus and nucleus ambiguus region of the brain, subsequently inducing parasympathetic nerve activation in the lung [12]. 

A very recent in vivo study indicated that chronic hyperinsulinemia in obesity can promote the sensory nerve population and enhance their ability to increase signal transduction to the brain in response to different stimuli in the airway [26]. High-fat-fed obese mice had hyperinsulinemia, leading to increased bronchoconstriction in response to vagus stimulus. However, selective depletion of insulin receptors on airway sensory nerves in these high-fat-fed obese mice prevented vagally induced bronchoconstriction. Furthermore, hyperinsulinemia in high-fat-fed obese mice was associated with increased length and branching of airway epithelia nerves and enhanced expression of neuronal substance P. However, these changes were prevented by depleting the insulin receptors on the airway epithelial nerves, indicating that increased insulin promotes bronchoconstriction by inducing hyperinnervation of sensory nerves in the airway [26].

Another study by Park et al., 2019, indicated that insulin resistance in high-fat-diet-fed mice can promote airway hyper-responsiveness that is associated with elevated transforming growth factor- β1 (TGF-β1) expression in their lung [27]. In addition, histological examination indicated increased collagen deposition in the perivascular and peribronchial areas, indicating lung fibrosis. Interestingly, the same study revealed that inhibition of TGF-β1-reduced airway hyper-responsiveness and lung fibrosis in high-fat-fed mice with or without OVA exposure. However, TGF-β1 did not affect airway hyper-responsiveness and allergic inflammation in OVA-exposed normal diet-fed mice. Furthermore, administration of insulin via the intranasal route for 11 days increased the expression of TGF-β1 in the lungs of normal diet-fed mice, which was also associated with lung fibrosis. Similarly, a significantly increased TGF-β1 expression was also observed in BEAS-2B bronchial epithelial cells in vitro after insulin exposure [27]. These findings inform that hyperinsulinemia in high-fat-diet-induced obesity can enhance airway reactivity and lung fibrosis by increasing the expression of TGF-β1 in the airway; however, further explorations are required in humans to confirm its clinical significance.

Various studies in humans have also indicated that insulin resistance in obesity plays a role in the development of asthma. In obese children, insulin resistance has been linked to an increased risk of allergic asthma, and increased waist circumference is associated with reduced forced vital capacity (FVC) and caused expiratory volume in one-second (FEV1) values in these children [28,51]. A retrospective study using a nationally representative dataset from the United States showed that insulin resistance progressively increases the odds of asthma incidence in obese individuals independent of hypertension, systemic inflammation, hyperglycemia, and hypertriglyceridemia [29]. Another retrospective cohort of obese asthmatic adults in the United States showed that prediabetes and diabetes state increases asthma exacerbation rate by 27% and 33%, respectively [30]. These studies underscore the role of insulin resistance in inducing airway hyper-reactivity in asthma and warrant a promising druggable strategy to reverse hyperinsulinemia for treating obesity-associated asthma.

## 5. Obesity, Inflammation, and Asthma

Rapidly growing adipose tissues of obese individuals promote a hypoxic environment and release various proinflammatory cytokines, including monocyte chemotactic protein 1 (MCP1) [52]. MCP1 can activate circulating monocytes, recruit them to adipose tissue, and induce their differentiation. Subsequently, these differentiated adipose tissue macrophages can promote local and systemic inflammation, leading to Th1 cell activation and release of Th1cell-mediated inflammatory cytokines [52]. Although obesity-associated asthma shows a signature of Th1-mediated inflammation, several studies have reported Th2-mediated inflammation, especially in obese children [53,54,55,56,57].

Increased levels of adipokines derived from adipose tissue mass in obese individuals also play an important role in asthma pathogenesis. An early study by Sideleva et al., 2012 showed that increased expression of obesity-associated adipokine, leptin, in the visceral adipose tissue of obese asthmatic women and that its enhanced levels are positively correlated with enhanced systemic inflammation and airway reactivity in these individuals without increasing airway inflammation [31]. However, several recent studies have shown that leptin can promote the secretion of different inflammatory cytokines in the airway cells by binding to its receptors on them [15]. For example, leptin induces the production of different cytokines and chemokines, including monocyte chemoattractant protein-1 (MCP-1), eotaxin, interferon gamma-induced protein 10 (IP-10), and interleukin (IL)-6 and IL-8, in normal human lung fibroblasts in vitro [32]. Hence, leptin can play an important role in promoting inflammation in the airway of asthmatic individuals by inducing the differentiation of fibroblast [32]. Another study indicated that leptin could promote the nuclear factor kappa light-chain enhancer of activated B cells (NF-κB) dependent expression of intercellular adhesion molecule (ICAM-1), human primary bronchial epithelial cells and the BEAS-2B airway epithelial cell line in vitro [33]. ICAM-1, a transmembrane glycoprotein, facilitates the infiltration of inflammatory cells, including eosinophils and neutrophils, into the lung during inflammation of the airway [58]. In addition to ICAM-1, leptin also promotes the secretion of various cytokines, such as C-C Motif chemokine ligand 11 (CCL11), vascular endothelial growth factor (VEGF), IL-6, and Granulocyte-colony stimulating factor (G-CSF) in BEAS-2B cells in vitro [33]. Although these studies collectively indicate that leptin has a proinflammatory role in promoting airway inflammation in vitro, future studies exploring the clinical significance of these findings need to be further explored.

Collectively, these findings indicate that increased adipose tissue and their mediators play several detrimental roles in inducing systemic and lung proinflammatory phenotype, and reducing adiposity and systemic inflammation might be a promising strategy to reduce airway inflammation in obese asthmatic individuals.

## 6. Metabolic Dysregulation in Obesity and Asthma

Metabolic dysregulation is a significant complication of obesity, proposed to contribute to different lung diseases central to obesity. Rautureau et al., 2021 reported that dietary obesity shows significant alterations in the metabolomic profile of the liver and lung of mice compared to other organs of the body, with the lung having an altered concentration of 1 carbon (1C) pathway and tricarboxylic acid (TCA) cycle metabolites, and increased triglyceride deposition [34]. A cross-sectional study by Liu et al., 2018 showed that obesity-asthmatic individuals have differentasthmaticic signatures in their serum, sputum, and peripheral blood monocytes as compared to those from lean asthmatic individuals. Pathway enrichment analysis indicated that these metabolites are involved in different pathways including cyanoaminoacid metabolism, alanine, aspartate and glutamate metabolism, pentose phosphate pathway, caffine metabolism, phenylalanine, tyrosine, and tryptophan biosynthesis, glycerolipid metabolism, and glyoxylate and dicarboxylate metabolism. In addition, correlation analysis indicated that these metabolites were significantly associated with airway inflammation, further highlighting the fact that metabolic dysregulation has a significant role in airway inflammation [35]. 

## 7. Obesity, Asthma, and Microbiome

Microbial niches located in various sites of the human body, such as the gastrointestinal tract, respiratory tract, and oral cavity, play a crucial role in shaping local and systemic immune responses, and dysbiosis in these niches is associated with the onset of various diseases including childhood asthma [59,60]. For instance, inadequate gut microbiome maturation during the first year of life in children of asthmatic mothers is associated with asthma at age 5 [61]. Similarly, a nested case–control study from Canadian Healthy Infant Longitudinal Development (CHILD) study found that children at increased risk of asthma are associated with transient dysbiosis of the gut microbiome, characterized by a reduced relative richness of the bacterial genera *Faecalibacterium*, *Lachnospira*, *Veillonella*, and *Rothia* in the first 100 days of life [62]. Another recent longitudinal study showed that a reduced abundance of bacterial taxa *Lachnospira*, *Lachnobacterium*, and *Dialister* in infants’ gut is associated with an enhanced risk of developing asthma at 5 years of age [63].

Recent studies indicate that microbiome-derived metabolites can impact the immune component in the local niche and distant organs [64]. For example, short-chain fatty acids (SCFA) produced by gut bacteria can promote an anti-inflammatory environment by directly binding to G protein-coupled receptors or inhibiting the epigenetic enzymes of histone deacetylase family in epithelial cells and immune cells residing in the gut [64,65]. However, the alteration in the levels of SCFAs is found to be linked with allergic diseases such as asthma. For example, children with a reduced level of fecal acetate, a most abundant SCFA, at 3 months of age are associated with atopic wheeze at 5 years of age [66]. Interestingly, feeding acetate has been shown to reduce airway inflammation in mice by increasing the numbers and function of T regulatory cells (Tregs) [67]. Other SCFAs, such as butyrate and propionate, can inhibit IgE-dependent or -independent degranulation and IL-6 production in mast cells derived from humans and mice [68]. Administering butyrate and propionate-producing species like *Akkermansia muciniphila* to mice reduces airway eosinophils and lung inflammation [36]. Gut bacteria such as *Morganella morganii*, *Lactobacillus vaginalis*, and *Escherichia coli* can release histamines that deleteriously affect lung health. Histamines can induce airway obstruction by promoting the contraction of airway smooth muscle cells, increasing mucus formation, and inducing airway mucosal edema [69,70]. 

Obesity is associated with gut microbial dysbiosis, reduced proportion of beneficial bacteria like the Bacteroidetes, and low bacterial diversity [71,72,73]. A recent meta-analysis reported that obese individuals have relatively lower ratio of *Bifidobacterium* and *Eggerthella* and increased proportion of *Acidaminococcus*, *Catenibacterium*, *Anaerococcus*, *Dialister*, *Escherichia*, *Shigella*, *Dorea*, *Eubacterium*, *Megaspera*, *Fusobacterium*, *Prevotella*, *Streptococcus*, *Roseburia* and *Sutterella* as compared to normal weight individuals [74]. Michalovich et al., 2019 indicated that obese asthmatic adults are associated with several alterations in immunological pathways and microbiota composition [36]. The study also found that specific microbial signatures in the gut are related to specific inflammatory markers in blood and bronchoalveolar lavage fluid (BALF) in obesity and asthma, and the presence of both diseases together has a synergistic impact on microbiome and inflammation [36]. Hence, modifying the microbiome in obesity-associated asthma might be a potential therapeutic approach to reduce immune dysregulation in asthma.

## 8. Diet in Obesity-Associated Asthma

Obesity is often associated with consuming a diet rich in fat and low in fiber. Early studies using a murine model have shown that a high-fat diet is associated with increased airway hyper-responsiveness, serum IgE, and lung TNF-α levels in OVA-challenged mice as compared to normal-diet-fed OVA mice, which was reversed with exercise or treatment with a normal diet [37]. The direct mechanisms linking a high-fat diet, airway inflammation, and hyper-responsiveness are not understood extensively. Hyperinsulinemia due to a high-fat diet increases airway reactivity and lung fibrosis by increasing TGF-β1 expression in the lung [27]. Furthermore, increased high-fat diet consumption leads to enhanced levels of saturated fatty acids (SFA) in the circulation. SFAs can alter innate immune response by activating different inflammatory signaling cascades such as NF-κB, ROS, and MAPK. Enhanced levels of circulating SFA have been reported in obese patients as compared to normal-weight asthma subjects [75]. Increased levels of SFAs have shown to promote NLRP3 inflammasome-dependent airway inflammation in asthma patients [75]. Recently, Dimasuay et al., 2023, investigated the impact of a high-fat diet and SFA, palmitic acid, on airway inflammation [38]. They found that the levels of palmitic acid and soluble dipeptidyl peptidase 4 (DPP4), a mediator of eosinophilic airway inflammation, are elevated in the BAL fluid of obese asthmatics as compared to lean asthmatics [38]. The authors also showed that high-fat-diet-induced obese mice have increased palmitic acid in the BALF and that these mice show increased eosinophils, neutrophils, and eotaxin-2 in the airway after IL-13 stimulation. The proinflammatory role of palmitic acid was further confirmed by treating IL-13- or HDM-exposed mice with palmitic acid, which showed increased eosinophils and neutrophils in the airway compared to the mice treated with either IL-13 or HDM [38]. Finally, the authors showed that palmitic acid exposure increases the release of exotoxin-3 and DPP4 in IL_13 pre-treated human bronchial epithelial cells in vitro, further confirming the proinflammatory role of palmitic acid in the airway [38].

On the other hand, replacing a high-fat diet with dietary fiber-rich food might have a beneficial impact in reducing airway inflammation in obese asthmatic individuals. Increased consumption of dietary fibers results in an elevated ratio of Firmicutes to Bacteroidetes in the gut of mice, resulting in increased levels of circulating SCFAs that eventually protect the mice from airway inflammation upon a house dust mite extract challenge as compared to their littermates that consumed a low-fiber diet [76]. A recent in vivo study by Wen et al., 2022, revealed that a diet rich in insoluble fiber also relieves airway inflammation in mice by increasing the composition of lipid-metabolizing bacteria without altering SCFA levels [77]. Collectively, these studies imply that a fiber-rich diet may play an important role in reducing asthma pathogenesis in obese asthmatic individuals and warrants further clinical investigations.

## 9. Current Management and Potential Future Therapies for Obesity-Associated Asthma

Obese-asthmatic individuals show reduced response to standard corticosteroid therapy due to obesity-specific factors such as non-T2 inflammation, mechanical changes in the airway, systemic inflammation, and microbial dysbiosis [78]. Since no approved pharmacological drugs are currently available for treating obesity-associated asthma, corticosteroid therapy remains the mainstay for asthma management in these individuals. According to the Global Initiative for Asthma (GINA) guidelines, weight reduction should be considered as an essential treatment strategy for obesity-associated asthma in addition to corticosteroid therapy. Furthermore, several randomized controlled trials and retrospective studies have informed that reducing weight by surgical intervention, diet, or exercise can help improve asthma symptoms and lung functions in obese asthmatic individuals [15].

Recent studies have investigated the potential benefits of reducing hyperinsulinemia in treating obesity-associated asthma (Table 2). For example, metformin, a classical oral hypoglycemic drug, is shown to have a beneficial role in treating asthma in murine models by reducing airway inflammation. Metformin treatment reduces lung infiltration of eosinophils, peribronchial fibrosis, reduced thickening of smooth muscle cells, and secretion of mucous in ovalbumin and fungal-associated allergenic protease challenged mice by enhancing AMPK levels and its phosphorylation [79]. In line with this finding, Ma et al., 2022, also showed that metformin-mediated activation of AMPKα enzyme reduces airway inflammation by decreasing the secretion of various cytokines including IL-4, IL-5, IL-13, tumor necrotic factor (TNF)-α, matrix metalloproteinase (MMP)-9, and transforming growth factor (TGF)-β1 into BALF and prevents the remodeling of airway by decreasing goblet cell hyperplasia, hypertrophy of airway smooth muscle cells, and collagen deposition in OVA-challenged mice [80]. Recent studies indicate that metformin also exhibits its pharmacological action on the gut and the liver [81]. Metformin treatment alters the gut microbiome by increasing SCFA-producing bacteria. For example, metformin increases the abundance of bacteria that produce butyrate, lactate, and propionate in type 2 diabetes patients. Relative abundance of Akkermansia, Lactobacillus, Bifidobacterium, Prevotella, Shewanella, Blautia, Megasphaera, and Butyrivibrio was increased in metformin-treated type 2 diabetes patients as compared to non-users [82,83]. Since SCFAs have shown anti-inflammatory effects in the airway, it is conceivable that metformin can have an anti-inflammatory impact in asthma patients by altering the gut microbiome.

Recently, several large cohort-based studies showed that metformin may be beneficial in reducing asthma symptoms in patients with concurrent asthma and diabetes. A retrospective study involving a cohort of 23,290 subjects with asthma and diabetes found that initiation of metformin treatment was associated with reduced asthma exacerbation and asthma-related hospitalization and emergency visits [84]. Another retrospective study, which examined the data from the Taiwan National Health Insurance Research Database over 11 years, found that diabetic asthma patients who used metformin had reduced asthma exacerbation and asthma-related hospitalization as compared to non-users [85].

Metformin reduces TNF-α-induced inflammatory signaling and NF-kB-mediated inducible nitric oxide synthase (iNOs) expression by restoring AMP-activated protein kinase (AMPK) activity in the lungs of high-fat-diet-induced obese mice upon ovalbumin (OVA) exposure [86]. Metformin treatment reverses weight gain in OVA-challenged high-fat-diet-induced obese asthmatic mice. The treatment also reversed the OVA-induced increase of IL-4, TNF-α, and decrease in IL-10 and IFN-γ in these mice, indicating its anti-inflammatory role in obese asthma. These changes were associated with increased number of regulatory T cells (Tregs) in the spleen of these mice [87]. Metformin also reduces non-allergic airway hyper-responsiveness in diet-induced obese mice. Here, metformin reduced serum insulin and methacholine-induced airway hyper-responsiveness without altering baseline airway resistance [88]. Similarly, another in vivo study by Calco et al., 2021, showed that metformin thwarts airway responsiveness in response to vagus nerve stimulation, inhibits weight gain and fat gain, and reduces insulin levels in diet-induced obese rats [89].

Similar to metformin, GLP-1 receptor (GLP-1R) agonists have also been shown to have beneficial effects on asthma. GLP-1R agonist treatment reduces the expression of IL-33 in bronchial epithelial cells in vitro and reduces its secretion into the BALF of BALB/c mice exposed to Alternaria extract challenge in vivo [90]. Consequently, GLP-1R agonist treatment also reduced IL-5 and IL-13 expressing innate lymphoid cells 2 (ILC2) cells in the lung and decreased the protein levels of cytokines and chemokines, including IL-5, IL-13, CCL17, CCL22, and CCL24 in BALF and lungs. These changes were associated with reduced perivascular eosinophilia, mucus secretion, and airway hyper-reactivity in Alternaria-extract-challenged mice compared to vehicle-treated mice [90]. GLP-1R agonist treatment has also shown a beneficial role in treating asthma in obese mice [91]. Treatment with a GLP-1R agonist reduces the secretion of epithelial alarmins, including IL-33 and TSLP, into the BALF and decreases the activation of lung ILC2 of Alternaria-extract-challenged obese mice compared to the vehicle treatment. In addition, GLP-1R agonist treatment also reduced the protein levels of Alternaria-extract-induced cytokines and chemokines IL-5, IL-13, CCL11, chemokine (C-X-C motif) ligand (CXCL)1, CXCL5, and neutrophil chemoattractants including keratinocyte-derived chemokines (KC) and lipopolysaccharide-induced CXC chemokines (LIX) in obese mice. There was also a noticeable reduction in the number of eosinophils and neutrophils in the airway of obese mice treated with the GLP-1R agonist. Methacholine challenge studies indicated that the GLP-1R agonist treatment dramatically reduced the protein level of IL-13, a marker of airway hyper-responsiveness in Alternaria-exposed obese mice compared to vehicle treatment. The same study also showed that the GLP-1R agonist reduces the expression of ICAM-1 in lung epithelial and endothelial cells of obese mice challenged with Alternaria extract [91]. In line with these findings, a study by Hur et al., 2021, also indicated that treatment with GLP-1R reduces eosinophilic inflammation and hyper-reactivity of the airway in OVA-challenged high-fat-diet-fed obese mice. Furthermore, the study also showed that GLP-1R antagonists could induce these changes by reducing the activation of NLRP3 inflammasome, caspase-1, and IL-1β [92].

A recent retrospective comparative study also indicated that GLP-1R agonist treatment reduces the number of asthma exacerbations in patients with concurrent asthma and diabetes, as compared to other anti-diabetic drugs such as sodium-glucose cotransporter-2 (SGLT-2) inhibitors, dipeptidyl peptidase-4 (DPP-4) inhibitors, sulfonylureas, or insulin [93]. However, no clinical trials have been performed so far on investigating the promising role of GLP-1R agonists in treating obese asthmatic individuals.

## 10. Conclusions

The relationship between obesity and asthma is intricate, and the exact mechanisms through which obesity contributes to worsening asthma are not entirely understood. However, recently, researchers have demonstrated that obesity-associated non-T2 factors have an essential role in promoting asthma pathophysiology by enhancing airway hyper-reactivity, airway inflammation and remodeling, and diminishing the response to standard asthma therapy. Given the link between obesity-associated non-T2 factors and asthma, this complex interaction requires further exploration to understand the molecular mechanisms governed by these factors in promoting asthma pathogenesis. For example, identifying the epigenetic factors and differential expression of immune-related genes due to the non-T2 factors will help us identify the molecular targets and develop a precision treatment for obese asthmatic phenotypes. Extensive understanding of molecular mechanisms involved in common T2 asthma has led to the development of successful targeted therapies recently. However, targeted therapies for obesity-associated non-T2 asthma are not yet available. Interestingly, in vivo and retrospective studies show that anti-diabetic drugs, including metformin and GLP-1R agonists, have great potential in treating obesity-associated asthma. However, no clinical trials of these drugs in obese asthmatic individuals have been carried out so far. Hence, future studies focusing on further understanding the molecular mechanisms through which obesity augments asthma severity and randomized clinical trials to evaluate the efficacy of metformin and GLP-1R agonists in the treatment of obesity-associated asthma are needed. 

## Figures and Tables

**Table 2:** Metformin and GLP-1R agonists in reducing asthma symptoms.

	In Vivo (Asthmatic Murine Studies) 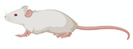	In Vivo (Obese Asthmatic Murine Studies) 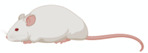	Retrospective Studies (Concurrent Asthma and Diabetes) 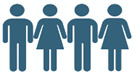
**Metformin**	↓ Lung infiltration of eosinophils, peribronchial fibrosis, smooth muscle layer thickening, mucus secretion, cytokine secretion in BALF	↓ TNF-α-induced inflammation and NF-κB-mediated iNOs expression in lungs ↓ Airway hyper-responsiveness, weight gain, fat gain, insulin levels data	↓ Asthma exacerbation ↓ Asthma-related hospitalization and ED visit
**GLP-1R agonists**	↓ IL-33 expression and secretion ↓ ILC2 activation ↓ Chemokines and cytokines ↓ Eosinophilia, mucus secretion, airway hyper-reactivity	↓ IL-33 and TSLP secretion ↓ ILC2 activation ↓ Chemokines and cytokines ↓ Eosinophils and neutrophils ↓ Airway hyper-reactivity ↓ ICAM-1 expression in lung epithelial and endothelial cells	↓ Asthma exacerbation

↓—decrease. Abbreviation: BALF: bronchoalveolar lavage fluid; IL: interleukin; ILC2: innate lymphoid cells type 2; TNF-α: tumor necrotic factor-α; NF-κB: nuclear factor kappa light-chain enhancer of activated B cells; iNOs: inducible nitric oxide synthase; TSLP: thymic stromal lymphopoietin; ICAM-1: intercellular adhesion molecule 1; and ED: emergency visit.

## Data Availability

Not applicable.
